# Editorial: Biosurfactants—A next generation biomolecules for enhanced biodegradation of organic pollutants

**DOI:** 10.3389/fmicb.2022.947801

**Published:** 2022-08-16

**Authors:** Punniyakotti Parthipan, Liang Cheng, Aruliah Rajasekar, Obulisamy Parthiba Karthikeyan, Pattanathu K. S. M. Rahman

**Affiliations:** ^1^Electro-Materials Research Lab, Centre for Nanoscience and Technology, Pondicherry University, Puducherry, India; ^2^School of Environment and Safety Engineering, Jiangsu University, Zhenjiang, China; ^3^Institute of Materials Engineering, Nanjing University, Nantong, China; ^4^Environmental Molecular Microbiology Research Laboratory, Department of Biotechnology, Thiruvalluvar University, Vellore, Tamil Nadu, India; ^5^Department of Civil and Environmental Engineering, South Dakota School of Mines & Technology, Rapid City, SD, United States; ^6^Institute of Bioresource and Agriculture, Hong Kong Baptist University, Kowloon Tong, Hong Kong SAR, China; ^7^Centre for Natural Products Discovery, School of Pharmacy and Biomolecular Sciences, Liverpool John Moores University, Liverpool, United Kingdom

**Keywords:** biosurfactants, surface tension, emulsification activity, optimization, organic pollutants, sophorolipids, bioherder

Microorganisms can produce a range of amphipathic molecules (containing both hydrophobic and hydrophilic moieties) that accumulate at interfaces between hydrophobic and hydrophilic phases. When biosurfactant accumulations reach threshold concentration “micelles” arise which act to reduce the surface and interfacial tensions to form emulsions. Biosurfactants differ greatly in their structures, which affects their specific functions/applications. Different types of biosurfactants are used in many industries/applications including detergents, pharmaceuticals, oil recovery, and bioremediation. Biosurfactants have numerous advantages over chemical surfactants in industrial bioprocess applications including (i) reduced toxicity, (ii) easily biodegradable, (iii) stable at higher pH and temperature, and (iv) result in greater foaming (Parthipan et al., 2017; Prakash et al., 2021). Furthermore, they can be synthesized using cheap raw materials such as agro-wastes and industrial waste materials that reduce the production costs and reduce waste disposal and associated environmental problems (Parthipan et al., 2021).

The focus of this Research Topic was to explore bioremediation applications of biosurfactants from hydrocarbon-degrading microbes. Deployment of biosurfactants in the bioremediation of hydrocarbon contaminants is gaining attention due to their significant outcomes, such as increasing bioavailability, effective removal efficiency, biocompatibility, reducing the uses of toxic chemicals (chemical surfactant), etc. (Rahman et al., 2003). Hydrophobic hydrocarbons (petroleum hydrocarbons and other environmental pollutants) can cause adverse effects on the environment as well as on human health. Many of these hydrocarbons are classified as toxic, carcinogenic, and mutagenic. The detrimental impact of these hydrocarbons is increased due to the difficulties in removing them from the environment (Lee et al., 2018). Conventional remediation methods such as chemical (chemical oxidation (with harmful oxidants), solvent extraction/washing), physical (incineration and *in-situ* thermal desorption), or biological (with ineffective microbes) methods have many limitations such as higher cost, slow process, and ineffectiveness (Hentati et al., 2021).

Biosurfactants can enhance the bioavailability of hydrocarbon molecules, thus improving hydrocarbon biodegradation. This Research Topic aimed to explore recent advancements in this research area with a focus on the identification of novel strains with effective biosurfactant producing capability and the impact of biosurfactants on the degradation of the different hydrophobic organic pollutants and heavy metal mitigation.

In the study “Identification of four secreted aspartic protease-like proteins associated with sophorolipids synthesis in *Starmerella bombicola* CGMCC 1576” by Liu et al. used site-directed deletion mutagenesis for the improvement of sophorolipids production by the yeast strain *Starmerella bombicola*. The sophorolipids production level was increased to 90% with 60.71 g/L in the strain Δ*sapl* after removing the coding genes clusters (*sapl1, sapl2, sapl3*, and *sapl4)* under the ammonium sulfate as a nitrogen source. However, no increase in sophorolipids production was observed in Δ*sapl* at yeast extract condition. Compared to that of the wild-type strain, the expression levels of the key genes for sophorolipids synthesis are upregulated in Δ*sapl* under ammonium sulfate conditions. Overall study, summarize that *sapl* gene cluster suppressed the key genes involved in the sophorolipids synthesis under ammonium sulfate condition by restraining the expression of the key genes involved in sophorolipids synthesis.

Optimization of the growth parameters could help microbes to produce their metabolites with the highest capability. A small change in their optimum growth level can hurt their activity. “Formulation of a culture medium to optimize the production of lipopeptide biosurfactant by a new isolate of *Bacillus* sp.: a soil heavy metal mitigation approach” saw Kalvandi et al. select lipopeptide-producing *Bacillus* sp. SHA302 strain and optimize biosurfactant production using the Plackett-Burman and response surface methodology. Different parameters such as pH, carbon source, and nitrogen source were optimized and obtained 5.74 ± 0.52 g/L of biosurfactant with 29.2 ± 0.71 mN/m surface tension activity. Increasing biosurfactant concentration leads to a decrease in the surface tension and finally achieving critical micelle concentration where micelles are formed. The highest lead and zinc release rate was observed (53.8% ± 1.4 and 39.3% ± 1.7, respectively) with the highest CMC with acid treatment. While increasing the concentration, heavy metals in adsorption sites form complexes (anionic biosurfactants form non-ionic complexes with metallic cations) with the biosurfactant and are further released into the soil solution.

A review paper published on this Research Topic “Biosurfactant: A Next-Generation Tool for Sustainable Remediation of Organic Pollutants” by Sharma et al. summarized the types of biosurfactants, the importance, and advantages of the biosurfactant on environmental applications. The detailed properties of the biosurfactant and their role in the bioremediation of organic pollutants are elaborated.

Oil spillage in marine environments are very common due to the widespread exploration and transport of crude oil from offshore locations. Safe burning is one of the effective methods to remove oil spillage. In a study “Bioherder generated by *Rhodococcus erythropolis* as a marine oil spill treating agent” by Yu et al. described that the addition of herding agents at the oil slick edges may swiftly diminish the oil-water interfacial tension (<1 mN/m) around the oil slick edges, so negative spreading coefficient was generated, which enables the thickening of the oil slicks to reach a new equilibrium. For effective burning, oil floating on the sea surface needs to be 2–3 mm thick to counter heat loss at sea and to provide sufficient oil vaporization to maintain oil burning. Even if biosurfactants are effective, their production cost becomes the major hurdle faced by the environmental industry for the development of bioherders. This study also highlights the fact that biosurfactant production costs can be decreased by utilizing low-cost substrates, such as waste and by-products (sugarcane molasses, steep corn liquor, and soy waste). Also, production costs could be further reduced through the development of engineered strains and innovative bioreactors.

As mentioned earlier, screening and identification of potential biosurfactant-producing strains will assist and make a significant impact on environmental clean-up technology. In a study, “polyphasic analysis reveals potential petroleum hydrocarbon degradation and biosurfactant production by rare biosphere thermophilic bacteria from deception island, an active Antarctic volcano” by Schultz et al. isolated 50 different thermophilic bacterial strains with biosurfactant production capability, and among them, 13 strains were effective emulsifiers.

Overall, these five different studies demonstrated the effectiveness and importance of biosurfactants in the bioremediation of organic and inorganic pollutants. The outcomes of this Research Topic clearly indicated how biosurfactants are important for environmental and industrial applications. The major drawbacks in biosurfactant usage are problems connected with their synthesis, including a lack of efficient microorganisms, higher production costs, and low yield. Hence, improvements in surfactant chemistry, biotechnology, and remedial technology are essential to the future application of biosurfactants.

## Author contributions

All authors listed have made a substantial, direct, and intellectual contribution to the work and approved it for publication.

## Funding

LC was supported by the program of Jiangsu Distinguished Professor, the Innovation/Entrepreneurship Program of Jiangsu Province, Jiangsu Province Key Project of Research and Development Plan (BE2020676), and the Initial Research Fund of Highly Specialized Personnel from Jiangsu University (4111370003). AR was supported by TANSCHE-RGP project (RGP/2019-20/TVU/HECP-0059). OP was supported by SD-EPSCoR SEED Grant. PR was supported by research funding from the Liverpool John Moores University and the Research England sponsored Expanding Excellence Fund to deliver transformative enzyme-enabled solutions for the circular recycling of plastics through the Centre for Enzyme Innovation at the University of Portsmouth.

## Conflict of interest

The authors declare that the research was conducted in the absence of any commercial or financial relationships that could be construed as a potential conflict of interest.

## Publisher's note

All claims expressed in this article are solely those of the authors and do not necessarily represent those of their affiliated organizations, or those of the publisher, the editors and the reviewers. Any product that may be evaluated in this article, or claim that may be made by its manufacturer, is not guaranteed or endorsed by the publisher.
